# Type I cryoglobulinemic vasulitis with eosinophilia: A case report and literature review

**DOI:** 10.1097/MD.0000000000016382

**Published:** 2019-07-12

**Authors:** Jingjing Wen, Fang Xu, Min Li, Qiaoling Zhou, Wen Qu, Yiping Liu, Jing Su, Hong Hu

**Affiliations:** a Hematology Department; b Rheumatology Department, Mianyang center hospital, Mianyang, China.

**Keywords:** cryoglobulinemia, eosinophilia, Monoclonal gammopathy of undetermined significance, purpura, vasculitis

## Abstract

**Rationale:**

Type I monoclonal cryoglobulinemia is usually associated with lymphoproliferative disorders, such as monoclonal gammopathy of undetermined significance (MGUS), myeloma, chronic lymphocytic leukemia (CLL) and lymphoplasmocytic lymphoma (LPL). Clinical symptoms related to Type I cryoglobulin (CG) isotype often include skin, neurological and renal manifestations.

**Patient concerns:**

A 42-year-old woman who initially presented urticaria, palpable purpura in both her upper extremities and legs, eosinophilia and Raynaud phenomenon. Skin biopsy revealed eosinophil infiltration. Monoclonal immunoglobulin (Ig) G-κprotein was detected and CG was also positive.

**Diagnoses:**

The patient was finally diagnosed as MGUS related Type I CG.

**Interventions:**

Cyclophosphamide-dexamethasone-thalidomide (CDT) therapy was initiated.

**Outcomes:**

The treatment relieved the skin symptoms efficiently.

**Lessons:**

To our knowledge, this is a rare case of Type I cryoglobulinemic vasulitis with eosinophilia complicated by MGUS, and the effective treatment of cyclophosphamide combined with thalidomide and prednisone may provide a new therapeutic option for cryoglobulinemic vasulitis.

## 1 Introduction

Cryoglobulins are referred to those blood proteins which precipitate at temperature lower than 37^o^C and redissolve on rewarming. Cryoglobulins are classified into 3 different categories. Type I cryoglobulinemia consist of monoclonal immunoglobulins or rarely monoclonal light chains in the serum.^[1]^ It is often related to lymphoproliferative diseases, such as monoclonal gammopathy of undetermined significance (MGUS), multiple myeloma (MM), Waldenström macroglobulinemia or lymphoma. Here, we report a case of Type I Cryoglobulinemic vasculitis (CV) associated with MGUS, which was distinctively featured by eosinophilia.

## 2 Material and methods

### 2.1 Ethical approval

This study was approved by the ethics committee of Mianyang center hospital, Mianyang, China Consent statement. The ethical batch number is P2019001.

Written informed consent was obtained from the patient for publications of this manuscript and accompanying images.

### 2.2 Case report

**Figure 1:**
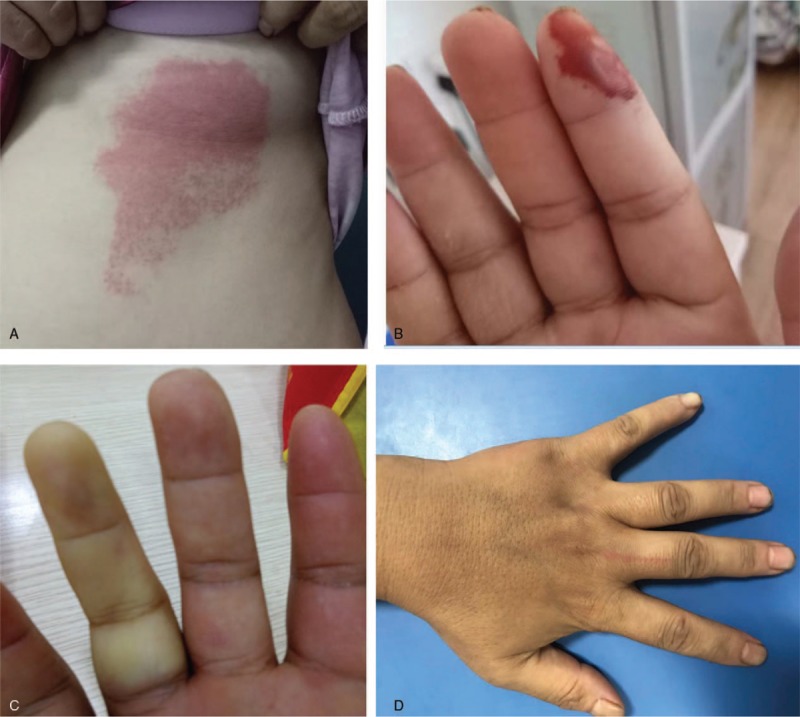
(A) Erythema on the skin of upper abdomen at initial stage. (B) Purpura on the fingertips. (C) Fingers turned pale encountering cold. (D). Vasculomotor phenomenon on the finger.

A 42-year old female was hospitalized for recurrent rashes in lower limbs and Raynaud phenomenon of fingers (Fig. 1). Initially, the rashes were itchy and the low extremities were involved. After the treatment of prednisone in local clinic, the rashes disappeared. Over time, the purpura of fingers, numbness in the limbs and Raynaud phenomenon developed. The rashes reoccurred due to discontinuation of prednisone.

On admission, the patient presented with purpura in the fingers, skin ulcer and edema around ankle. She also complained pain of digits. The skin biopsy was performed in center of erythema on the right lower limb. Microscopy showed lots of infiltration of eosinophils and some of lymphocytes.

Complete blood count revealed eosinophilia [1.54 × 10^9^/L, normal range (NR) 0.02–0.52 × 10^9^/L]. Uterine protein was negative and renal function was normal. Other results were negative including hepatitis viral, HIV, antinuclear antibody, rheumatoid factor assays and antineutrophil cytoplasmic antibody. No hepatosplenomegaly was found by color Doppler ultrasonography. Electromyogram of upper extremities was normal.

**Figure 2:**
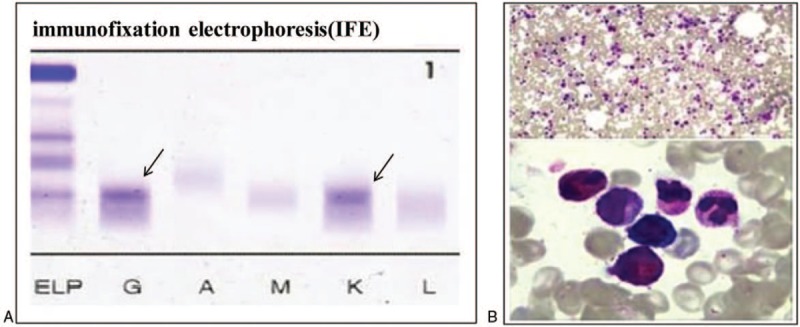
(A) Immunofixation electrophoresis showed a monoclonal IgG-light kappa chain. (B) Bone marrow smear examination showed an increase of eosinophils (19.5%).

Initially, the patient came to the department of rheumatism in our hospital. Eosinophilic panniculitis was suspected and prednisone was administered. The rashes disappeared and the pain in digits relieved. But the patient still complained recurrent numbness and purpura of fingers especially in cold environment. Based on eosinophilia, the patient was investigated by a hematologist. Also hypogammaglobulinaemia was noted with immunoglobulin (Ig) A 705 mg/L (NR 836–2900 mg/L) being below normal. Further laboratory tests as follows were applied. No obvious monoclonal gamma spike was found by serum protein electrophoresis. Immunofixation electrophoresis showed a monoclonal IgG-light kappa chain (Fig. 2 A). Bone marrow smear examination showed an increase of eosinophils (19.5%) (Fig. 2 B). Flow cytometry identified existence of clonality in plasma cells (1%) with aberrant expression of CD56. Skeletal X-ray and spinal MRI were negative. β2 microglobulin was 1.431 mg/L (NR 0.9–2.0 mg/L). Type I cryoglobulins were detected at 4^o^C. Thus, cryglobulinemia associated with MGUS, complicated with secondary eosinophilia, was diagnosed. Then, the patient was treated with compound cyclophosphamide at a dose of 50 mg on day 1 to 4 and predisone at a dose of 60 mg on day 1 to 4 every 28 days. Prophylactic warming and cold avoidance was also advised. After 4 cycles of treatment, the symptoms relieved and the cryoglobulin (CG) could not be detected.

## 3 Discussion

In this brief report, we describe a case of MGUS-related CV with cutaneous involvement and eosinophilia. Cryoglobulins are immunoglobulins that precipitate in vitro at temperatures less than 37°C and redissolve after rewarming. Cryoglobulinemia refers to the presence of cryoglobulins in serum. Three basic types are recognized according to the clonality and type of immunoglobulins. Type I consists of monoclonal Ig (IgM, IgG, or IgA), as well as monoclonal free light chains. Type II cryoglobulins are a mixture of monoclonal IgM and polyclonal IgG. Type III cryoglobulins are a mixture of polyclonal immunoglobulins of all isotopes (mostly IgM and IgG). Types II and III are referred to as mixed cryoglobulinemias because they consist of both IgG and IgM components. The term CV is used to describe patients with symptoms related to the presence of cryoglobulins.^[1]^ This patient was verified as Type I cryoglobulinemia by immunofixation electrophoresis. We summarized the literature and see Table 1.^[2,3,5,7,14–17,20]^

**Table 1:**
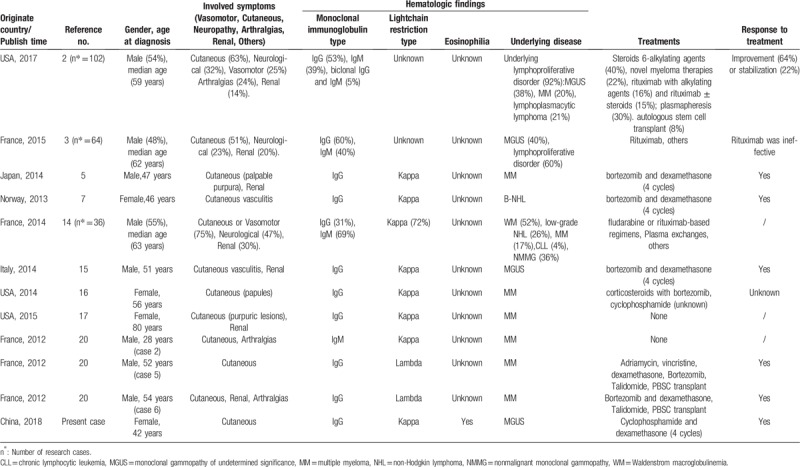
Summary of clinical characteristics in patients with type I cryoglobulinemia and cryoglobulinemic vasulitis with skin involvement.

Usually cryoglobulinemia may lead to lesion of skin, injury on kidney, arthritis and peripheral neuropathy. Vasculitic purpura is the most common symptom. In 89 cases of symptomatic cryoglobulinemia Mayo clinic reported, cutaneous symptoms were found in 63% patients, 43.42% with ulcers or gangrene.^[2]^ Harel et al summarized 64 patients diagnosed with Type I cryoglobulinemia, cold-triggered ischemic skin manifestations were observed in 33 patients 51%). Cutaneous involvement variably combined with Raynaud phenomenon, livedo reticularis, recurrent episodes of cold-induced necrotic purpura of the extremities and urticaria, either cold-related or not.^[3]^ Thirty patients with cryoglobulinemia from Peking union medical college hospital were analyzed by Shi Xiao-hu et al.^[4]^ It showed 66.7% and 63.3% of the patients were accompanied with purpura rash and chronic renal insufficiency.^[4]^ Skin complications were also frequently referred by many other case reports.^[5–9]^ Similar as reported, cutaneous symptoms in this patient included rashes, Raynaud phenomenon and vasculitic purpura either.

Eosinophilia could be associated with a variety of malignant neoplasms, especially hematopoietic neoplasms, including Hodgkin disease non-Hodgkin's lymphoma of T-cell lineage and etc. However, eosinophilia related to plasma cell myeloma is rarely reported.^[10]^^.^ Literature review showed eosinophilia was rarely combined with cryoglobulinemia and MGUS either. The distinctive character of our patient was eosinophilia. And eosinophils infiltration was also found by skin biopsy. We tried to find other possible causes that could explain eosinophilia, but failed.

Some studied have verified eosinophils were related to the pathogenesis of solid tumors.^[11–12]^ But, the exact mechanisms proposed to explain the occurrence of peripheral blood eosinophilia in patients with plasma cell myeloma are unknown. Stefanini et al^[13]^ have suggested 4 hypotheses. First, release of a protein from necrotic tumor cells may result in eosinophilia. Second, the presence of metastatic tumor in the bone marrow may stimulate eosinophilopoiesis. Third, eosinophilia may represent a genetic-related familial response to malignant tumors. Finally, the neoplastic cells may actively secrete substances that either directly, or indirectly via secretion by other cells, result in eosinophilia. Glantz et al^[10]^ reported a case of plasma cell myeloma and marked peripheral blood eosinophilia, 109.7  × 10^9^/L. And the authors believed IL-3 secreted either by the neoplastic cells at a level below detection by immunohistochemistry or by other cells in response to the presence of plasma cell myeloma may have played a role in causing the eosinophilia.^[10]^ It may probably explain the increase of absolute eosinophil count in the case of MGUS-related cryoglobulinemia.

Type I monoclonal cryoglobulinemia is usually associated with lymphoproliferative disorders or plasma cells diseases. So far, it is clear that this case is associated with MGUS, but the further underlying disease of this case is still unknown. In the serial cases Harel et al^[3]^ reported, 26 in total of 64 patients with Type I monoclonal cryoglobulinemia (40%) were considered to have a MGUS whereas 38 patients (60%) had an overt lymphoproliferative disorder. The 38 patients with an IgG CG were initially diagnosed with MGUS (n = 18), MM (n = 13), lymphoplasmocytic lymphoma (LPL) (n = 2) and chronic lymphocytic leukemia (CLL) (n = 5).^[3]^ As Mayo Clinic reported majority of the underlying diseases were lymphoplasmacytic lymphoma, especially Waldenström macroglobulinemia and MM.^[2]^

As far as Ig isotype concerned, IgG and IgM seemed to be more common in Type I monoclonal cryoglobulinemia. Harel et al^[3]^ reported that the 64 monoclonal cryoglobulinemia were of the IgM or IgG isotype in 26 (40%) and 38 (60%) cases, respectively. In 94 cases Mayo Clinic reported, thirty-nine patients (38%) had underlying MGUS, of which 25 (64%) were IgG, 12 (31%) were IgM and 2 (5%) patients had biclonal IgG and IgM MGUS.^[2]^ Also, many other case reports showed Type I monoclonal cryoglobulinemia with IgG-Kappa, the same as our patient.^[5,7,14–17]^

There are no treatment guidelines for cryoglobulinemia due to the rarity of the disease. The clinical severity of Type I cryoglobulinemia is linked to related symptoms, the organs involved and the underlying diseases. Literature review indicates most treatments were based on underlying hematologic malignancy and therapeutic target. Sidana et al^[2]^ retrospectively analyzed the most cases of cryoglobulinemia from Mayo clinic. Treatment regimens consisted of steroids with alkylating agents, rituximab with alkylating agents, rituximab ± steroids, plasmapheresis and autologous stem cell transplant.^[2]^ It is similar as Antoine Néel et al reported^[14]^ in indolent lymphoma or MGUS associated Type I cryoglobulinemia, polychemotherapeutic regimens containing rituximab and/or fludarabine seemed to be more effective than single alkylating agent or single agent rituximab.^[14]^ For those patients with severe skin necrosis, renal manifestations and systemic presentation, repeated complete plasmatic exchanges were recommended, in combination with various chemo- and steroid-based regimens.^[3]^ Bortezomib is another promising treatment choice for Type I cryoglobulinemia, even after treatment failure of rituximab.^[7,18–21]^ Initially our patient took prednisone and the rashes disappeared. As the diagnosis of Type I cryoglobulinemia was established, prednisone, cyclophosphamide and thalidomide were used. The patient's cutaneous symptoms relieved and absolute peripheral eosinophil count was normal. Considering there was no other system organs involved, no further treatment was applied.

Type I cryoglobulinemia is an uncommon clinical disorder. It is rarer to be combined with eosinophilia. Cyclophosphamide combined with thalidomide and prednisone is an effective treatment to be recommended for cryoglobulinemic vasulitis. Although MGUS was diagnosed, it is still unclear how the patient will progress and should be followed up continually.

## Author contributions

**Conceptualization:** Fang Xu, Yiping Liu.

**Investigation:** Min Li, Qiaoling Zhou, Wen Qu, Yiping Liu, Jing Su, Hong Hu.

**Writing – original draft:** Jingjing Wen.

**Writing – review & editing:** Jingjing Wen, Fang Xu.
